# Control and Ownership of Neuroprosthetic Speech

**DOI:** 10.1007/s13347-019-00389-0

**Published:** 2020-01-22

**Authors:** Hannah Maslen, Stephen Rainey

**Affiliations:** Oxford Uehiro Centre for Practical Ethics, University of Oxford, Suite 8, Littlegate House 16/17 St Ebbe’s Street, Oxford, OX1 1PT UK

**Keywords:** Brain-computer interfaces, Neuroprosthetics, Control, Ownership, Responsibility, Speech

## Abstract

Implantable brain-computer interfaces (BCIs) are being developed to restore speech capacity for those who are unable to speak. Patients with locked-in syndrome or amyotrophic lateral sclerosis could be able to use covert speech – vividly imagining saying something without actual vocalisation – to trigger neural controlled systems capable of synthesising speech. User control has been identified as particularly pressing for this type of BCI. The incorporation of machine learning and statistical language models into the decoding process introduces a contribution to (or ‘shaping of’) the output that is beyond the user’s control. Whilst this type of ‘shared control’ of BCI action is not unique to speech BCIs, the automated shaping of what a user ‘says’ has a particularly acute ethical dimension, which may differ from parallel concerns surrounding automation in movement BCIs. This paper provides an analysis of the control afforded to the user of a speech BCI of the sort under development, as well as the relationships between *accuracy*, *control,* and the user’s *ownership* of the speech produced. Through comparing speech BCIs with BCIs for movement, we argue that, whilst goal selection is the more significant locus of control for the user of a movement BCI, control over process will be more significant for the user of the speech BCI. The design of the speech BCI may therefore have to trade off some possible efficiency gains afforded by automation in order to preserve sufficient guidance control necessary for users to express themselves in ways they prefer. We consider the implications for the speech BCI user’s *responsibility for* produced outputs and their *ownership of* token outputs. We argue that these are distinct assessments. Ownership of synthetic speech concerns whether the content of the output sufficiently represents the user, rather than their morally relevant, causal role in producing that output.

## Introduction

Implantable brain-computer interfaces (BCIs) are being developed to restore speech capacity for those who are unable to speak. Patients with ‘locked-in syndrome’, amyotrophic lateral sclerosis, or other communication-impairing pathologies could be able to use covert speech – vividly imagining saying something without actual vocalisation – to trigger neural controlled systems capable of synthesising the speech they would have spoken, but for their impairment.

The importance of user control has been identified as particularly pressing for this type of BCI. The incorporation of machine learning and statistical language models into the decoding process introduces a contribution to (or ‘shaping of’) the output that is beyond the user’s control. Whilst this type of ‘shared control’ of BCI action is not unique to speech BCIs (Glannon 2016; Tamburrini 2009; Tonin et al. 2010), the automated shaping of what a user ‘says’ has a particularly acute ethical dimension, which we argue differs from parallel concerns surrounding automation in movement BCIs.

In this paper, we offer an analysis of the loci and limits of user control over speech BCIs, with implications for the user’s degree of moral responsibility for outcomes causally facilitated by the BCI. In doing so, we argue that the ethical importance of users’ control intersects with conceptual and empirical questions regarding the *accuracy* of the output and the user’s *ownership* of the output. We therefore address these related areas in order to assess the full moral relevance of control over neural speech prostheses. We consider the implications for the speech BCI user’s *responsibility for* produced outputs and their *ownership of* the content of those outputs. We argue that these are distinct assessments and provide criteria for ownership of synthetic speech.

## Overview of the Technology for Speech Prostheses

Electroencephalogram (EEG) controlled devices have been under development for some time. These devices read brain signals externally from the scalp (typically) in order to provide input to devices, or software, thereby giving control to an EEG device user. The sort of control they offer is limited but sufficient for tasks like moving a cursor on a screen or moving a prosthetic limb through space. A prosthesis designed to produce continuous speech will require a particularly high degree of control, at least where the aim is to facilitate conversational speech in all its complexity. Research and development of these complex speech prosthesis systems is ongoing (Bocquelet et al. 2016b; Brumberg et al. 2010; Guenther et al. 2009; Martin et al. 2014). To illustrate the mechanisms involved, we focus on one example of this sort of technology.

BrainCom is a multidisciplinary European project aiming to develop neuroprosthetic technology for speech.^1^ The technology is designed to pick out, process, and decode linguistically relevant neural signals so that the speech that these signals represent can be externalised artificially. This raises the possibility of realising speech for those who may have lost their ability to communicate through, for example, disease or injury.

Previously existing technologies, such as EEG, are somewhat effective at recording neural signals relevant to speech. However, they operate too slowly and generally at too low a resolution to be of use in a realistic speech situation, such as conversation. Progress in electrocorticography recording has provided the means to surpass these limitations. Via probes placed directly onto the cerebral cortex, high-resolution electrical activity can be read from the brain very quickly. These probes can be placed in important regions of the cortex related to speech, such as the motor areas associated with mouth and throat movements.

Taking advantage of novel materials and device designs, BrainCom is developing new microelectrocorticography (μECoG) technology to decode articulatory-related activity from specific cortex areas. These brain regions are associated with the planning of movement, movement itself, and linguistic expression. When users engage in ‘covert speech’ – when they *imagine producing words clearly in their head* – the articulatory motor cortex realises patterns of activity as if they were actually vocalising words.

**Fig. 1:**
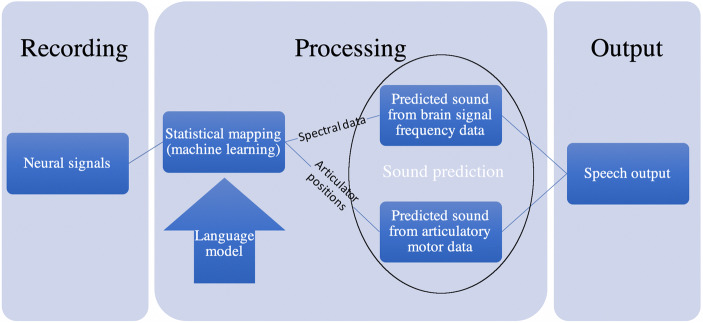
This illustration shows essential parts of the process from input of neural signals to speech output. Neural signals, in this case correlates of covert speech, are recorded and prepared for processing. The actual covert speech activity of the user constitutes a variable input to this stage, but not a random one, as only specific signals from known brain areas are recorded (e.g. spectral features, articulatory motor data). This allows processing of the signals in terms of how given signals correspond with probable speech outputs and with positions of the lips, tongue, velum, etc. This is bolstered with a model of language that further constrains processing according to rules (such as likelihood of one syllable following another). Likely syllable combinations can be predicted from the language model. Combinations of syllables, forming words, can also be predicted based on learning from the actual covert speech activity of a user: predictions based on ‘fixed’ rules of a language can be adapted according to the actual covert speech behaviour of a user. Machine learning can predict likely speech outputs based on prior speech outputs, as well as a language model. This processed neural and language information can then be input to a vocoder in order to output synthetic speech

From the information recorded using ultra flexible, high-density μECoG electrodes, speech could eventually be ‘decoded’ with very high accuracy. This is based upon inferences from neural activity in the articulatory motor area to the phonemes that the articulatory movement would produce, as reconstructed by machine learning algorithms. ‘Appropriate processing and decoding’ could involve the deployment of ‘a statistical language model (giving the prior probability of observing a given sequence of words in a given language)’ (Bocquelet et al. 2016a n 7, 396) (Fig. 1).

This kind of model aids word prediction and ought to optimise the system ‘to capture important syntactic and semantic information of language […] by calculating probabilities of single words and probabilities for predicting words’ (Herff et al. 2015). This gives the system a predictive function, offering faster and more reliable brain signal decoding. However, the more a system *interprets* brain signals in processing them, or the more predictive the system becomes, the potential for a ‘gap’ between user speech intention and speech output arises (Rainey 2018). This makes an analysis of accuracy, user control, and speech output ownership important.

## The Relationship Between Accuracy, Control, and Ownership

Although we are principally interested in assessing user *control* of neural speech prostheses and implications for users’ responsibility, we must also attend to related considerations that relevantly bear on control – what it involves and why it is important. In this section, we explain why we will need to clarify what we should mean by *accuracy* for these purposes, as a principally descriptive consideration relevant to the assessment of whether the user is in principle able to reliably generate an output that corresponds to the input. This is a mechanistic consideration pertaining to a dimension of controllability of the device; accurate functioning of the device is necessary for reliable control. We also explain that an assessment of the importance of user control prompts consideration of the extent to which, and frequency with which, the user is able to generate outputs for which she takes *ownership* – outputs that she endorses and identifies with as her own. This is, in part, an ethical consideration, pertaining to the user’s ability to use the device to produce satisfactory outputs.

An assessment of accuracy of a BCI is, in broad strokes, an assessment of the concordance between the input and the output. For instance, the relative accuracy of predicting a vowel sound given the signals recorded from an intracortical microelectrode (Bocquelet et al. 2016a, p. 396; Mugler et al. 2014). When accuracy is assessed in this sense, an assessment is made of the degree of ‘match up’ between one thing and something else that represents it. By way of analogy, when the accuracy of a map is assessed, the evaluative focus is on the extent to which it tracks, in its representation, the features of a space. When the accuracy of someone’s throw is assessed, the evaluative focus is on the extent to which the trajectory of the ball – where it is located in space – matches up with the target location in space. When the accuracy of a language translator is assessed, the focus is on the extent to which the meaning of the original is preserved in the translated version.

All of these examples involve questions of concordance between one thing and another to provide an assessment of accuracy. Control and accuracy are related. For some types of engagement with the world, greater control will lead to greater accuracy. For example, the greater the control I have over the movements of my arm, the more accurately I will be able to hit the target with the ball. For other types of engagement, the greater the accuracy of the device, the more control afforded to the user. For example, the greater the accuracy of the map, the more control the map reader has over whether they will arrive in the place they need to be. In the case of the translator, the user has no control over the translation once their input text has been entered; they are completely dependent on the accuracy of the translator to generate the concordant output. The point of these examples is to show that control and accuracy are related, and that in some cases accuracy affords greater control, in other cases greater control affords greater accuracy, and in others control and accuracy are somewhat independent.

The relationship between control and accuracy in the case of a speech BCI will be complex and will roughly involve all three types of relationship outlined above;

- The user will have some control over the continuous input:
  - They control their covert speech activity
- They will learn to *work with* the way the BCI represents (or ‘maps’) the input, learning to adapt their input accordingly
  - Their training in and experience with their device will bolster their control
- But they will also lack control over much of the processing that contributes to the output
  - Brain signal processing will be at least partly algorithmic, according to rules of a language model and machine learning prediction

We elaborate on how we should conceive of the accuracy of speech BCIs below in section 4. For now, it will be useful for subsequent sections to elaborate a little on the second point above concerning user training, as it bears heavily on control. Training involves (1) familiarising the user with the way that the device works (roughly, giving them knowledge of how to use the device), (2) practice, to cultivate the user’s ease of use of the device (roughly, developing their ability to control the device), and (3) training of the system itself as it learns to map user-specific inputs to outputs (roughly, improving accuracy of the system).

Training has been described as one of the most important but least understood aspects of BCI use in general (Wolpaw et al. 2002, p. 780). The onerous nature of BCI operation can make prolonged use difficult. Training for the use of a neural controlled prosthetic limb can take weeks which, whilst impressively quick, is nevertheless a significant endeavour (Farahany 2011).

The kind of training required to use a BCI that recreates speech based on articulatory motor brain signals is multidimensional. As the user becomes familiar with the system and learns to control it, the device, too, undergoes training. In particular, a deep neural net (DNN) can learn to process neural signals in terms of their likely developments, given a language model. This allows prediction of likely articulatory motor brain activity, given likely concurrent phonetic phenomena in spoken language:

The DNN was trained on a dataset of [electromagnetic-articulography] and audio recordings simultaneously acquired from a reference speaker. Once trained, this DNN was then able to convert the movement trajectories of the tongue, lips, jaw and velum into continuously varying spectral parameters, which, in turn, could be transformed by a vocoder to generate a continuous speech waveform (with a proper excitation signal). (Bocquelet et al. 2016, p. 398)

Being trained in using a BCI system for speech therefore includes the user becoming familiar with and practiced in using the system. It also includes the system optimising probabilities concerning likely neural activity given various learned parameters.

Training is highly important for user control. Important dimensions concern the user’s interaction with the device and the voluntariness and precision of their causal contribution to the process of producing speech. Relevant questions include whether a user is able to control the neural activity taken as the input, how voluntary this is, and whether the user is able to determine when the device is active. Such assessments of user control also concern the extent to which the user contributes in a precise and intentional way to the overall activity of the device to produce an output. Whilst such assessment will have normative implications, for example, for responsibility, the assessment of user control concerns mechanisms.

Control and accuracy bear on the user’s relationship to the output of the device – the synthesised speech in the case under consideration. We characterise this as the user’s ownership of the synthesised speech and will explain below that this amounts to the user’s endorsement of the content therein. Whilst increased accuracy and control should lead to greater ownership of the output, how we evaluate the sufficiency of the user’s ownership of their speech will require more than asking whether the device is accurate or the user has control; it will require an account of ownership. Given the necessarily limited amount of control the user will have, in the light of the contribution made by the device via decoding and processing, the most pressing ethical question will be whether the user sufficiently endorses the output; control is important in so far as it facilitates this.

Following the analysis of each concept below, we will argue that the relationships between accuracy, control, and ownership in this context can be glossed as follows: control *via* accuracy *for* ownership.

## Accuracy of the Device

As outlined above, assessing accuracy requires determining the degree of concordance between two things. In this section, we introduce two senses of accuracy that pertain to BCIs for speech: technical accuracy and semantic accuracy. We argue that an assessment of technical accuracy will apply to speech BCIs in a way similar to BCIs for movement. However, the assessment of semantic accuracy of a speech BCI is difficult, whereas its counterpart for movement BCIs is more straightforward.

### Technical Accuracy

Technical accuracy pertains to the good functioning of the neuroprosthetic system, from the recording of neural activity through processing to the production of an acoustic output. In this narrow sense, ‘accuracy’ means that there is a close correspondence between neural activity, the recording made by the neuroprosthetic device, the digital representation of the recording, and the synthetic speech output, in a pairwise fashion. Assuming the device is accurate for each successive pair, the final output will be accurate with respect to the initial input. Notice that accuracy can obtain between different modes of representation: in the same way that the map represents geographic space, or a musical note represents musical sound, the device will represent the neural activity as a digital signal, which will ultimately be decoded to translate it into speech (also discussed in Rainey et al. 2019).

Therefore, the entire neuroprosthesis could be called ‘accurate’ if it:

- Accurately detects and records neural activity associated with an instance of covert speech
- Accurately represents the neural activity digitally
- Accurately maps the representation of the neural activity to corresponding articulatory motor properties
- Accurately derives acoustic properties from the articulatory motor properties
- Accurately produces a synthetic verbal output matching those acoustic properties

Technical accuracy is necessary in order to be able to use the device in a reliable way. It is required in order for the user to have any control over what is generated. However, when we assess the accuracy of the device, we will be interested in more than its degree of technical accuracy. This is partly because the most important outcome, at least from the point of view of using the device, is that the device can be used to say what the user intended to say. Call this semantic accuracy. Further, we should ask this independent question because technical accuracy will not guarantee semantic accuracy. There is a contribution to the process that is not simply a matter of accurately representing information in one medium in an alternative. The statistical language models and algorithmic prediction employed in the processing to some extent transform the information to impose meaning where it is lacking, or correct where indeterminate or highly improbable (Bocquelet et al. 2016b; Lei et al. 2016; Song and Sepulveda 2014). Thus, even though there are many stages of the process where accuracy can be assessed – accuracy that will facilitate control – there are other stages where information is transformed or introduced by the process. This will happen, for example, when the user does not generate a perfect input, such that technical accuracy alone is not sufficient to result in semantic accuracy.

### Semantic Accuracy

We now flesh out this notion of semantic accuracy. We have said that accuracy involves concordance. In the case of technical accuracy, accuracy roughly involved information being gathered, represented, mapped, and represented again. Such accuracy will concern objective facts about the neural activity of the user, the way the machine operates at each stage, and the way the information gets converted. In the case of semantic accuracy, the relevant concordance is between what the user wanted to say and the content of the synthetic speech. Whilst this makes intuitive sense – i.e. that the user can assess whether the device said the single word or short phrase he or she indented to say – semantic accuracy becomes more difficult to operationalise in the context of the continuous speech developers hope these devices will enable.

Whilst there will be a ‘fact of the matter’ regarding the technical accuracy of many of the computations made by the device, some argue there may be no equivalent fact of the matter for what we are calling semantic accuracy (Carruthers 2013). Indeed, in instances of natural speech, what a speaker says often does not quite match what they attempt to say, and speakers sometimes seem to ‘find out what they want to say’ through speaking. Sometimes speakers express themselves more eloquently than they thought they could, or might use a word they have never used before, perhaps even being surprised at this word use. The point is that whilst speakers usually know whether they have expressed themselves in a way they are happy with, they sometimes cannot be sure whether what they said is what they meant to say. That is, it is not clear whether what they say is ‘accurate’ because there is no independent, fully formulated linguistic content against which to compare it. Rather when we engage in continuous speech we have what we call ‘hazy intentions’ with respect to our speech.

Hazy speech intentions can be conceptulized as underspecified or abstract intentions regarding the content of intended speech. The use of this term aims to capture the phenomenology of intending to express oneself without knowing yet exactly how one will do so. An intention to agree with an interlocutor, to commend a point of view, to dispute a point, or anything similar, can be acted on decisively by an agent, even when she lacks a specific plan for the words she will select. In this way, hazy speech intentions are not opaque to the speaker, who is often very aware that she intends to express herself in some way. Rather, hazy speech intentions are hazy in the sense that they lack specificity regarding word use. Speakers often have a general goal when they start to speak, but specific words are selected as speech is implemented.

We shall elaborate on the distinction between clear and hazy intentions below. At this point though, the key claim that we want to make is that semantic accuracy of natural, continual speech is difficult to assess precisely because of the hazy nature of these intentions. This difficulty will be compounded in the case of BCI-mediated speech. This is, again, because reconstruction and prediction algorithms, partly using statistical language models, may ‘fill in blanks’ or resolve indeterminacy. The device will, in a sense, be estimating what the user is trying to say. The more the device departs from what the user intended to say, as opposed to the model ‘smoothing’ speech that would otherwise be poorly expressed, the more semantic accuracy would be jeopardised. Even with these possibilities in mind, the user may still not be sure whether the device altered the output.

### Clear vs. Hazy Intentions and Instrumental vs. Expressive Goals

At this point it will be instructive to compare this difficulty in assessing semantic accuracy with the parallel assessment of accuracy of BCIs for movement. This will illuminate further the haziness of intentions regarding speech and demonstrate some preliminary implications for control. A number of authors have discussed the kinds of control that users are likely to have over BCIs for movement (Glannon 2014; Haselager et al. 2009; Steinert et al. 2018). Such BCIs include neurally controlled robotic arms and brain-actuated wheelchairs, amongst other devices (Güneysu and Akin 2013; Leeb et al. 2015; Schalk et al. 2004; Tamburrini 2009). Of particular note is the contribution the device makes to the movement performed. It is important at this point to clarify the way in which the user shares control of the device, which in turn makes predictions about what the user intends to do. This is because the clarity of the user’s intentions will affect the prospects for intention-concordant action, partly controlled by the device.

One example of a neurally controlled robotic arm involved implanting a 4 x 4 mm electrode array into the part of the participant’s motor cortex that is associated with the dominant hand (Hochberg et al. 2012). The decoder was calibrated using the participant’s recorded neural activity during a training phase in which the participant simultaneously watched and imagined controlling a robotic arm as it moved. Ultimately, the participant’s neural activity, produced as they imagined moving the robotic arm, could be decoded to control the arm. However, for a complex task such as picking up a bottle of coffee and drinking from it, parameters on movement were imposed, and the participant’s decoded grasp state was used as a sequentially activated trigger for one of four different hand actions that depended on the phase of the task and the position of the hand. The device predicted the intended action and robust finger position, and grasping of the object was achieved by automated joint impedance control.

Brain-actuated wheelchairs provide another example of shared control and predicted intention (Galán et al. 2008; discussed in Tamburrini 2009). The user of the wheelchair can issue four basic commands – forward, stop, left turn, and right turn – by engaging in four different mental tasks that generate very different patterns of neural activity, detected via EEG, and which are distinguishable by a classifier. However, these ‘raw’ commands can be combined with the perceptual state of the robot (free space, obstacle, wall left or right) to generate ‘shared commands’ in the case of a wall, which the chair will ‘follow’, or independent commands in the case of an obstacle, which the robot will autonomously avoid. Fully usable examples of this kind of technology would also fill in for lapses in the user’s commands.

We shall now elaborate on our aforementioned distinction between hazy and clear intentions, which we suggest has significant implications for devices that make predictions regarding user’s goals and supplement user control. In contrast to the haziness of our intentions regarding our speech, we far more often have quite clear intentions regarding our movement, or at least, regarding the overall goal of our movement. The user of a BCI arm might intend to pick up a cup so they can drink from it or, engaging in more complex action, might intend to use the arm to pack objects into their bag. The user of the brain-actuated wheelchair might intend to move from one side of a room to the other, or to board a bus. Since these goals are more clearly defined, it will be much easier to determine whether a goal has been precisely fulfilled. The analogue of semantic accuracy in the case of BCIs for movement becomes a question of precision of goal fulfilment: how closely does the eventuating state of affairs match the intended state of affairs? This can be assessed much more clearly. We suggest that this is partly a consequence of movement often being entirely instrumental, whereas speech is inherently expressive and indirectly (and often secondarily) instrumental.

We will argue below that this difference has implications for how we think about specific dimensions of control over BCIs for movement and BCIs for speech. Goal selection, an aspect of executory control (defined below), is more instinctive and feasible in the case of movement, and evaluation of success in achieving movement goals is more clear-cut. There will be a clearer fact of the matter regarding movement goal fulfilment as compared to semantic accuracy (expressive goal fulfilment). This, we will argue, has implications both for the relative importance of this type of user control and for the need to substitute more objective assessments of sufficiency for more subjective assessments when it comes to speech BCIs, which cannot rely on an independent estimation of semantic accuracy.

## Control

As explained above, understanding the control the user has over the workings of the BCI involves assessment of the user’s interaction with the device and the voluntariness and precision of their causal contribution to the process of producing synthetic speech. Given the contribution made to the output (or act) by the device, users will not have full control. In this section we follow others (Steinert et al. 2018) in distinguishing between different dimensions of control, as relevant to the processes under consideration.^2^ This facilitates careful analysis of the aspects over which users exert control, and how fine-grained this control is.

### Control Over ‘that Something Is Spoken’: Executory Control

The first feature of control we must consider is the user’s control over the event that *something* is said. Asking whether the user has this control is equivalent to asking whether and with what degree of regularity the production of synthetic speech at a given time is voluntary. We might also think of this as the user’s ability to choose whether and when to use the device.

This acting or not acting – control over when and whether one acts – requires what has been called ‘executory control’. Steinert et al. (2018, sec. 4.3.1) describe executory control as follows:

People have many desires, beliefs, and intentions on which they do not act. Something additional has to come in to realize such intentions: an executory command. Often, it is called a volition. But because the term is controversial and ambiguous, we rather speak of an executory command. From a commonsensical perspective, the idea seems plausible: Sometimes, it occurs to us as if we give a conscious command, a go-signal, to initiate an action, and that it causally executes the action.

We can, at least theoretically, separate two aspects to this notion of executory control. The first is the simple go-signal – that *something* is initiated, the second is the *particular action* selected for initiation. In standard cases of action, practically, the two are not separable. We cannot, as it were, command ourselves to perform a ‘potluck’ act. As Steinert et al say, we command *an* action. In the case of BCIs, however, these aspects of executory control may be more practically separable, so we may need to consider the *mere commanding* of the BCI, as well as the ability to command specific acts.

This will be highly important for speech BCIs. It will be critical to avoid instances of unintentional speech: if a private thought or mind-wandering moment were to generate brain activity sufficiently similar to that produced when engaging in covert speech, the device could record, decode, and ultimately make overt this instance. If this aspect of executory control were not afforded to the user, the voluntariness of their BCI-mediated speech would be significantly compromised.

Whether or not the user of the speech BCI is afforded this dimension of control – the go-signal – is the first question, the answer to which will depend on the ability of the device to distinguish between voluntary engagement in covert speech and something like the ‘inner thoughts’ or private inner monologue of the user. It is clear that this aspect of control will be crucial.

### Control Over ‘What Is Spoken and How It Is Spoken’: Executory Goal Selection and Guidance Control

Determining *that* an instance of synthetic speech occurs is not the only dimension of control the user would want to have. In order to use the device to express themselves, users will also need to have control over *what* is spoken and *how* it is spoken. This shifts the focus to the second aspect of executory control: the action commanded or goal selected. It also introduces a second species of control: guidance control^3^, which is required in order to govern implementation – how an act or goal is pursued. This latter species of control also shifts the mechanical focus from the initiation of the process to the process itself.

Steinert et al. describe guidance control as follows (2018, sec. 4.3.3):

Sometimes, after initiation, people have control over the ensuing execution of movements, which is the ability to alter and influence the execution of movements, such as the trajectory of a limb movement […] Guidance control is often limited. For instance, we are not aware of the many muscles and their contractions that are necessary to raise an arm, let alone to perform more sophisticated actions such as skiing. Overall, humans lack fine-grained muscular control. Nonetheless, through various feedback channels, we monitor the progression of our movements and are able to adjust them, i.e., we could get them under conscious control, although only on general levels.

We can see that the importance of guidance control in the possibility it affords the user to intervene on and shape the action. We will argue that this particular aspect of control is more important for speech BCIs than BCIs for movement, although we will acknowledge that reasons of efficiency will direct away from simply maximising this type of control.

It seems in the case of speech BCIs, the user will need to be able to exert significant guidance control. That is, it will need to be continuously possible for the user to alter and influence the phrases produced. This is a consequence of there not being a clear goal that the user can command in the case of speech. The device will not be able to identify a command to say particular sentences independently from the user forming the words (although it might make predictions). However, the user will not have complete guidance control – over each word or even phrase spoken – due to the algorithmic prediction and correction that occurs as part of the processing.

This particular importance of guidance control for speech BCIs and apparent inseparability of the goal (what is to be said) from the process (saying what is to be said) is in contrast to motor BCIs. In the case of motor BCIs, the most important dimension of control will relate to carefully specified goal selection – whether these are more basic goals (turn left) or more complex goals (pick up object in front of me). As argued above, goal specification for continuous speech is not feasible – the content of the speech to be said becomes apparent through (covertly, and therefore also synthetically) speaking it.^4^

The difficulty of identifying goals for speech independently of formulating the speech (at the least, engaging in covert speech) constitutes a mechanistic limitation that justifies emphasis of guidance control for speech BCIs. However, there is a further reason generated by the generally differing purposes of speech versus movement. As we suggested above, speech is expressive and indirectly instrumental, whereas movement is principally instrumental. Whilst this will not always be the case,^5^ the preponderance of instrumental purpose for movement means that, very often, the process is less important to the user than fulfilling the goal. For example, with certain examples of motor BCI, the user will not care very much how the device moves in order to achieve the goal, as long as it does so in an efficient and unproblematic way. The user of a brain-actuated wheelchair, for example, may not mind which precise path the device takes to reach the other side of the room. Indeed, they may be particularly happy to offload this aspect of control, since the device may be able to operate more smoothly without the user exerting moment-to-moment guidance control. As noted above, the brain-actuated wheelchair discussed by Tamburrini (2009) includes two low-level behaviours– ‘obstacle avoidance’ and ‘smooth turning’ – that are governed by a behaviour-based robotic controller. That the user does not have to provide the commands for these herself makes it easier for her to move around space as she wishes (assuming she wants to avoid obstacles and walls).

Although there may be some cost in terms of sense of agency, with respect to motor BCIs, as long as the goal can be precisely defined, it will often be better if parts of the movement are on autopilot. Too much guidance control would compromise the user’s ability to achieve the goals she intends. Indeed, this reduction in guidance control may even increase agents’ global autonomy. In directing the BCI, the user still makes meaningful decisions, but the execution of the goals the user decides to pursue is rendered smoother and requires less effort.

In contrast, in the case of speech BCIs, users do care how the ‘goal’ is reached and ‘process’, or expression, is often more important than precise goal fulfilment. In parallel to our comments on semantic accuracy, it may not even be clear what the goal is. However, as outlined, devices for both speech and movement will share control of much of the process, making predictions about the user’s intended speech or actions.

### Comparison with Predictive Texting

It is instructive to compare the speech BCIs we are discussing with a potentially parallel, and far more familiar, example of prediction in our communicative efforts. It might be thought that the systems we described are relevantly similar to software for predictive texting (Thurlow and Poff 2013). In this case, too, errors can be made, and prediction can go awry. However, there are a number of differences, which also serve to highlight the potential importance of the final type of control for speech BCIs.

We suggest that the individual using predictive text software has much more control over what is communicated than the user of a speech BCI, notwithstanding the algorithmic intervention in predictive text. First, the texter is far more aware of the ‘workings’ of the process than the speech BCI user: the texter can see the words as they are produced and corrected. Although corrections to words typed may occur automatically, often the texter is able to determine whether to select the next predicted word, which is not inserted automatically. Further, the words appearing in ‘draft’ are not the end of the process and do not constitute the act of communication. Taken together, these features of texting offer an opportunity to reverse predicted words or reject corrections, and the texter is able to gauge when a predicted or corrected word is the word they intended to type. As noted, the drafting of the text is not the act of communication itself, and although corrections and predictions can still be missed, the texter needs to make an *additional* go-signal – pressing send on the text – in order for it to transform into an act of communication. Unlike the speech BCIs envisaged, texting as communication does not occur in real time, whereas synthetic speech, when the user engages in it, will be continuous, without a ‘drafting’ stage. Finally, as we will explain in further detail below, this real time ‘hearing oneself’ could make it more difficult for the user to ascertain what they intended to say versus what the device may have predicted or corrected.

### Veto Control: Additional No-Go Command

The above comparison shows that, although predictive texting can generate confusion in cases in which the texter does not pay attention, they do have a window to reject the contribution made by the algorithm. The requirement of an additional go-command makes this possible. Whilst this will not be possible for the user of a BCI for continuous speech,^6^ an alternative mode of control could be enabled, which would allow the user to stop the production of speech in its tracks, halting the process.

This kind of control has been called veto control (Clausen et al. 2017, p. 1338; Mele 2009, p. 51ff; Steinert et al. 2018, sec. 4.3). Conceptually, there may be cases of standard action in which the control exerted is identical with process control: if I stop my arm just before I touch what I suddenly realise will be a hot stove, I exert control over the process that, until that point, had propelled my arm towards the stove. The question about whether veto control is distinct from process control is a matter of debate depending on how one views the mereology of processes (Mele 2009), and in cases where an agent has fine-grained and precise process control over an action, there may be a case for not drawing a distinction. However, in the case of a speech BCI (or, indeed, a BCI for movement) where more of the process is automated, it would make more sense to think of a veto command (and the control it affords) as distinct from ceasing or redirecting the process.

Regardless of the correct conceptualisation of the user’s intervention via a ‘no-go’ command, such a possibility would be valuable, allowing the user to retract synthetic speech as it was spoken. The more automated the process, the more valuable this will be.

### Control : Preliminary Conclusions

To summarise the discussion of control so far, we have argued that technical accuracy facilitates (but does not guarantee) a high degree of control. Additional technical features, such as clear discrimination between neural activity associated with covert speech versus neural activity associated with mere thoughts, will be required to prevent unintentional vocalisation and maintain executory control.

We have also argued that better guidance control is likely to improve semantic accuracy (in so far as this can be assessed) and will facilitate ownership of speech. This is because the greater control the user has over the speech generated, the less it will be shaped by the device. However, we will now argue that even maximising guidance control will not in all cases guarantee ownership of the speech produced. Further, we have acknowledged that greater user control may come at the expense of efficiency, and so efficiency and sufficient ownership of speech may need to be carefully balanced.

In turning now to consider ownership of synthetic speech, we shift the importance from controlling the device or process itself to the user’s relationship to its outcomes.

## Ownership of Synthetic Speech vs. Responsibility for Producing It

The final dimension to consider concerns the relationship between the user of a speech BCI and the output. There are at least two types of relationship that we might consider important. The first, as we introduced above, is the user’s ownership of the synthetic speech that constitutes the output. The second is the user’s responsibility for the output.

These, we will argue, are separate questions. The question of whether the user has ownership of the speech relates to an assessment of whether the speech sufficiently represents the user, their views, feelings, or wishes. In contrast, questions regarding responsibility require (roughly) assessment of the degree of control the user had and the foreseeability of the outcomes for any given input (see Glannon 1997). The focus in the BCI literature has been on responsibility for harm caused whilst controlling a BCI, such as a wheelchair. For example, Grübler (2011: 380) suggests: ‘Given that a user is skilled and familiar with a device, we can reshape the problem in terms of reliability [of the device] and responsible use. By doing so, the focus shifts from a single and detached occurrence to a practice of using. And being responsible for this practice means dealing with risks in a (morally) responsible manner.’

We agree that responsibility for harm caused via neuroprostheses should be thought of as harm resulting from the use of a complicated and sometimes risky tool. Glannon (1997) emphasises that full control is not necessary for moral responsibility. The agent’s knowledge of the limits of their control and the probability of certain outcomes eventuating are also morally relevant. Correspondingly, the conditions for moral responsibility – of sufficient control and/or knowledge of probabilities of outcomes, even in the absence of control – could be present such that the user is responsible for having produced a speech output. However, we will argue that the user’s moral responsibility for harm (or good) in such an instance does not entail that the content of the output is ‘hers’ in the way we would be concerned about when evaluating someone’s utterance as really representing their view, feelings, or desires. We return to responsibility and comment on the implications of this below in 6.1.

Understanding what ownership of a BCI output consists in is particularly pertinent in the case of BCIs for speech where the primary purpose is often to express *oneself* and only indirectly serving an instrumental purpose. We should here distinguish what we are calling ownership from the phenomenological experience of agency. Users of a robotic arm can experience a sense of ‘estrangement’ with respect to an act, whilst simultaneously being satisfied that the device was used to act as they intended (Gilbert et al. 2017). Steinert et al. refer to this as a ‘missing sense of agency’ and a ‘peculiarity’ of BCI-mediated action (2018, sec. 3). Feeling estranged from the BCI-mediated act concerns the user’s experience of their sense of agency and not necessarily their endorsement of the act (or anything analogous to its ‘content’). The user’s well-being could be diminished through such experiences of estrangement, and it will be important not to neglect this. However, the question of ownership of the content of speech concerns more than whether the user feels alienated from the BCI and its operation in the world.

In addition to ownership of speech being distinguishable from a sense of agency, notice that ownership of speech is also not equivalent to, nor guaranteed by, semantic accuracy (defined above). Semantic accuracy, where possible to evaluate, *is not sufficient* for ownership of speech. This is because there are plausible instances of word-for-word externalisation of speech that the user does not own. For example, intrusive thoughts, practice thoughts, or ‘verbal thinking’ in general (Alderson-Day and Fernyhough 2015) might be externalised such that the speech is semantically accurate without the user identifying with the content. Interestingly, even in standard cases of speech, speakers sometimes ‘disown’ what they said, as in cases where people immediately retract their speech: ‘I don’t know why I said that’ or ‘that’s not what I meant to say at all’.

Further, semantic accuracy *is not strictly necessary* for ownership of speech (although increasing the rate of semantic accuracy, in so far as this could be possible, would facilitate ownership). To illustrate that semantic accuracy is not necessary for ownership, consider an instance in which a prosthesis user does not plan in detail what she is going to say, or an instance in which there is significant shaping by processing of the device, yet the user identifies with and endorses the output. In natural speech cases, we sometimes plausibly take ownership of content that we did not directly produce: ‘you said it better than I could myself’ or ‘you took the words out of my mouth’. Particularly where speech is produced via an algorithm that has learnt what the user tends to say, this could be a plausible possibility: *past authorship and endorsement could be sufficient for present ownership*, even if the speech were predominantly predicted by the BCI in the present instance.

So far we have argued that, although a higher degree of executory and guidance control over a technically and semantically accurate system will facilitate ownership of speech, it will not guarantee it. However, given comments above regarding standard cases of agents disowning their speech and clarifying their expressive intentions, it might be thought that there is nothing particularly different or challenging in the BCI case (assuming high enough levels of control and accuracy). It might be argued that we are setting the bar too high for BCI-mediated speech, given that standard speech contains errors, and agents sometimes retract or disown what they say. We agree that it is important not to overstate concerns. However, we suggest that there are two related ways in which BCI-mediated speech differs from standard speech that make it important to have an account of the conditions for ownership of synthetic speech.

The first is the contribution that the algorithmic processing and prediction makes to the output, absent in standard cases. The second concerns the influence of ‘hearing oneself’ saying things on one’s evaluation of what one wanted to say, especially where this may have been shaped by the device. Speakers sometimes take their cue about what they want, believe, or feel from hearing themselves say things – they think through speaking. For BCI-mediated speech, there may be some parallels here with the psychological phenomenon of ‘verbal overshadowing’ (Alderson-Day and Fernyhough 2015, p. 939; Perfect et al. 2002, p. 973). Unlike standard speech, the device may, in a sense, ‘suggest’ content, although the user experiences the speech as originating from herself (further demonstrating that sense of agency, discussed above, is distinct from ownership). This, we argue, makes it particularly important to develop an account of ownership of synthetic speech. We now outline a sketch of an account.

### A preliminary Account of Ownership of Synthetic Speech

We do not have space to develop a full account of ownership of synthetic speech here. However, our analysis above suggests a few key features, on the basis of which we can provide a preliminary sketch. In sketching an account, we also further distinguish ownership of synthetic speech from responsibility for synthetic speech and from phenomenological ‘sense of agency’.

There is existing work that relates to the concept of ownership of BCI action and its relationship to responsibility. Discussing an example of a neurally controlled robotic arm, Farahany (2011) claims that ‘[b]rain-machine interface enables us to [...] isolate separately the intention to act, the action, and identification with the action’. We agree that these aspects of engaging with the world are indeed more clearly separable in cases of BCI-mediated action than in cases of standard action. Farahany discusses these aspects in relation to implications for freedom of action, which she suggests have further implications for responsibility for BCI action. She argues that:

Freedom of action requires all three [intention, action, and identification]. Without proper identification with an action, a disjunction arises between the actor’s intention and the resulting action. Identification requires both subjective alignment by the actor with the resulting action and alignment by objective onlookers between the actor and the action. If an actor identifies with an action that an objective onlooker rejects as the actor’s own (e.g. because of facts known to the onlooker and unobservable by the actor), then attribution of responsibility is unwarranted. The disjunction undermines the presumption of the action as objective indicia of the actor’s subjective intent. And praise or blame would be misplaced upon an actor who does not properly identify or appreciate the action as his own.

We do not have space to discuss these claims in detail. However, we note that Farahany seems to claim that subjective alignment (perhaps equivalent to ‘sense of agency’) is necessary but not sufficient for freedom of action (and therefore responsibility). We assume that the ‘objective onlooker’ is party to facts about the causal history of the action, including facts about the user’s control and knowledge; facts that must obtain in order for the actor to be responsible, regardless of whether the action is experienced by the actor as originating from her intention. We agree that subjective alignment alone (assuming it corresponds to sense of agency) is not sufficient for responsibility. Particular causal and epistemic conditions must also be met.

Farahany also seems to claim that subjective alignment is necessary for responsibility. That is, where the relevant causal facts obtain but there is no sense of agency on the part of the actor; the actor will not be responsible. Whilst we agree that the absence of sense of agency might ‘undermine the presumption’ that the action is a product of the actor’s intentions, we do not think it would be impossible, particularly given the unusual mode of action that BCIs permit. Lack of sense of agency should prompt interrogation of facts regarding causal history, control, and user’s knowledge, but it would not be impossible that a user might knowingly control a device (if imperfectly) to engage in an action from which he feels estranged. Instances of this were seen in work conducted by Gilbert and colleagues (Gilbert et al. 2017).

This being said, we do agree that that there might be some plausible doubts regarding the actor’s *blameworthiness* for some aspects of a BCI-mediated action. We will proceed to argue for precisely such a doubt. Particularly when it comes to speech, we might question the user’s blameworthiness in connection to the particular content of the speech, as distinct from their blameworthiness for producing that instance of speech. However, we suggest that the relevant consideration is the user’s ownership of the content, rather than their sense of agency over its production. Whilst these might be collapsed in the case of motor BCIs (the outputs of which usually do not contain clear content), they are particularly separable for speech BCIs. Whilst sense of agency concerns the user’s phenomenological experience of acting, ownership, as we conceive it, concerns rational endorsement of content.

We now present our account of ownership. Our account is subjective. Semantic accuracy, were it to be objectively verifiable, would not ground ownership. As argued above, agents could coherently disown semantically accurate speech, and, particularly pertinent for BCIs, past authorship and endorsement could be sufficient for present ownership. Semantic accuracy is not necessary or sufficient for ownership.

A more plausible account of ownership of synthetic speech is as follows:

- If the user were to engage in critical reflection on the content of an instance of synthetic speech in the light of its BCI-mediated etiology (i.e. knowing that it will have been influenced in various ways by the device), she either:
  i) would endorse it as coherent with her character, as constituted by her diachronic values, desires, and beliefs (including as evidenced by past instances of communication), or
  ii) could provide alternative reasons for her endorsement of the content of the speech, especially if it represents a change in, or inconsistency with, some previous aspect of her values, desires, or beliefs. i.e. she could render her endorsement intelligible.^7^

Notice that the emphasis is not on whether the user caused the speech in a certain way but rather on whether she endorses it and could, if asked, provide reasons for taking ownership of the speech. It is sufficient for ownership that the speech simply reflects character (this makes conditions for ownership not unduly demanding). As with standard speech, owned speech does not have to be the product of significant thought on the part of the user, and the user can take ownership of device-predicted habitual phrases, for example.

However, *where the output is not coherent with the user’s character*, in order for ownership to obtain the user would (at least hypothetically) be able to provide further reasons for endorsement, as simple coherence would not explain or ground ownership in such instances.

It is important to note that our account of ownership is conceptual. Practical assessment of whether synthetic speech represents the user’s wishes in, for example, important decision-making contexts would need to err on the side of caution. Procedures to verify users’ wishes may therefore set the bar higher. However, being sufficiently sure of ownership is not the same as ownership obtaining *per se*.

Having clarified what we take ownership of speech to involve, we can now more clearly distinguish it from questions of responsibility. Given the different criteria for ownership compared to responsibility, we argue that users of speech BCIs might be morally responsible for a token instance of synthetic speech without being blameworthy for the particular content uttered.

This would especially be the case where the device contributes to the speech in a way the user did not even hazily intend (but where they foresee that the device sometimes deviates in some way) or where the device externalises a fleeting thought, not endorsed by the user and not intended to be externalised (again where such an eventuality is foreseeable). So, even if the user were responsible for a token of harmful utterance, this alone does not guarantee that they own or identify with its content. The user might therefore be blameworthy for allowing the device to produce a harmful utterance, where this is the product of lapse of control or aberrant decoding (where the possibility of aberrant decoding is sufficiently foreseeable), without the user being blameworthy for the particular content of that utterance. To be clear, we do not suggest that blameworthiness and moral responsibility come apart here. Rather, there are two separable objects for which a user is potentially morally responsible: a user can be morally responsible (and therefore blameworthy) *for producing* a token of speech utterance with a BCI and yet not deserve blame in response to *the particular content* of that token of utterance.

## Conceptual Insights and Technological Implications

We have argued that technical accuracy will facilitate (but not guarantee) control, which in turn facilitates (but does not guarantee) ownership. Through comparing speech BCIs with BCIs for movement, we have argued that, although goal selection is the more significant locus of control for the user of a movement BCI, control over process will be more significant for the user of the speech BCI. The design of the speech BCI may therefore have to trade off some possible efficiency gains afforded by automation in order to preserve sufficient guidance control necessary for users to express themselves in ways they prefer. Our analysis has a number of implications for developers:

- Developers should ensure that a speech BCI affords the user as much executory control as possible, with a focus on building in a go-command or reliably distinguishing between covert speech and inner monologue, such that the user controls *that* something is spoken.
- Some possible efficiency gains of automation must be traded off with enough of user’s own contribution to the process to increase guidance control. This allows the user to better control *how* speech is produced. This is important as, in the context of continuous communication, there is no way for the user to issue a command regarding what is to be spoken independently from engaging in the real-time covert speech.
- Ensuring that algorithms do not, as it were, take on life of their own will increase the prospects for the user’s ownership of the synthetic speech output. However, given the necessity of processing, developers should also make veto control possible to stop speech in its tracks.

Beyond implications for the development of this specific type of technology, our analysis has broader relevance for how we assess responsibility for action mediated by a BCI and how we understand the relationship between the user of a BCI and her action. We argued that a BCI user’s moral responsibility for action and its effects could in some cases be assessed separately from the extent to which the action or output represents or ‘belongs to’ the agent. This is due to the hazy nature of speech intentions, combined with the dimension of shared control. This assessment of ownership is particularly relevant in the context of speech, since speech is often expressive of aspects of the user’s character and values. Our paper raises broader philosophical questions about whether this dual assessment – of responsibility for vs. ownership of speech – is appropriate also in instances of standard speech. In standard cases of speech, we perhaps more readily assume that a retracted or unendorsed utterance nonetheless revealed something about the speaker (and so the speaker can still be blamed in relation to the content). Determining whether this assumption is justified will require examination of the degree and nature of agents’ control over standard speech, in comparison to neuroprosthetic speech. Further, our discussion raises questions about the norms and communicative practices that should be adopted in communicative contexts where speech is mediated via technology (Rainey et al. 2018; Kreitmair 2019).

## Footnotes

1 http://www.braincom-project.eu/

2 In referring to executory control and guidance control (as well as to veto control), we follow the language used by Steinert et al. However, we are aware that others use different terms (e.g. Shepherd 2014, 2015, who distinguishes between the executive and implementational dimensions of control). Any differences implied by the use of different terms do not affect our analysis; the terms employed by Steinert et al. capture the important distinctions we seek to illuminate.

3 A distinction is worth making here between this sense of ‘guidance control’ we use and the sense employed in the compatibilist case argued by Fischer and Ravizza. Their distinction is not about goal setting versus implementation; rather, it is about actual causal control (guidance control) and contra-causal freedom (regulative control) as they seek to demonstrate the compatibility of moral responsibility with lack of contra-causal freedom.

4 If goal specification were to be an appropriate part of analysis in speech BCI, it would be at a very different level than for motor BCIs. It would either involve a rapid series of concrete micro goals relating to production of syllables or at most words, or an underspecified macro goal to, for example, relay a series of facts about one’s day. With motor BCIs, there are much more obvious, highly specifiable macro goals.

5 For example, sign language and dancing will be examples of expressive movement.

6 Parallel functionality could be designed into a speech BCI if speech were not to be continuous. The device could provide the user with a chance to listen to a ‘draft’ via headphones, requiring them to issue an additional go-command before the speech is played via a speaker and allowing them to delete the draft via an alternative command.

7 For a related view on assessing the ‘true self’ in the context of personality changes accompanying therapeutic deep brain stimulation, see Pugh et al. (2017).
